# eTRANSAFE: Building a sustainable framework to share reproducible drug safety knowledge with the public domain

**DOI:** 10.12688/f1000research.74024.1

**Published:** 2022-03-07

**Authors:** Sirarat Sarntivijai, Niklas Blomberg, Katharina B. Lauer, Katharine Briggs, Thomas Steger-Hartmann, Johan van der Lei, John-Michael Sauer, Richard Liwski, Miranda Mourby, Montse Camprubi, The eTRANSAFE Consortium

**Affiliations:** 1 ELIXIR Hub, Wellcome Genome Campus, Hinxton, Cambridge, CB10 1SD, UK; 2 Lhasa Limited, Granary Wharf House, 2 Canal Wharf, Leeds, LS11 5PS, UK; 3 Bayer AG, Research & Development, Pharmaceuticals, Investigational Toxicology, 13342 Berlin, Germany; 4 Department of Medical Informatics, Erasmus University Rotterdam, EUR - Erasmus Medical Center (MC), Rotterdam, The Netherlands; 5 Predictive Safety Testing Consortium, Critical Path Institute, Tucson, Arizona, 85718, USA; 6 Centre for Health, Law and Emerging Technologies (HeLEX), Faculty of Law, University of Oxford, Oxford, OX2 7DD, UK; 7 Synapse Research Management Partners S.L., C. Diputació 237, Àtic 3a, 08007, Barcelona, Spain; 8 UK Research and Innovation, Polaris House, North Star Avenue, Swindon, SN2 1FL, UK

**Keywords:** FAIR Data, Research reproducibility, Interoperability, eTRANSAFE, Drug Safety, model validation, data sharing

## Abstract

Integrative drug safety research in translational health informatics has rapidly evolved and included data that are drawn in from many resources, combining diverse data that are either reused from (curated) repositories, or newly generated at source. Each resource is mandated by different sets of metadata rules that are imposed on the incoming data. Combination of the data cannot be readily achieved without interference of data stewardship and the top-down policy guidelines that supervise and inform the process for data combination to aid meaningful interpretation and analysis of such data.

The eTRANSAFE Consortium's effort to drive integrative drug safety research at a large scale hereby present the lessons learnt and the proposal of solution at the guidelines in practice at this Innovative Medicines Initiative (IMI) project. Recommendations in these guidelines were compiled from feedback received from key stakeholders in regulatory agencies, EFPIA companies, and academic partners. The research reproducibility guidelines presented in this study lay the foundation for a comprehensive data sharing and knowledge management plans accounting for research data management in the drug safety space - FAIR data sharing guidelines, and the model verification guidelines as generic deliverables that best practices that can be reused by other scientific community members at large.

FAIR data sharing is a dynamic landscape that rapidly evolves with fast-paced technology advancements. The research reproducibility in drug safety guidelines introduced in this study provides a reusable framework that can be adopted by other research communities that aim to integrate public and private data in biomedical research space.

## Introduction

In recent years, the need for data sharing and large-scale data integration has emerged in many domains, including life sciences and systems pharmacology, due to the benefit of investigating multidimensional perspectives with complex analyses. The Innovative Medicines Initiative (IMI) eTRANSAFE Consortium is a public-private collaboration between academic research institutes and EFPIA partners established to build an analytical platform for drug safety model validation by connecting various pre-clinical and clinical data types through linked-data and computational pharmacology approaches. Data integration in modern medicine and pharmacology requires careful consideration for research sustainability and reproducibility commencing in the planning stage. Implementing the eTRANSAFE analytical platform draws private data from the consortium partners, and combines these with open data from public resources, to identify the requirements for building a framework of sustainable and reproducible knowledge sharing through the dialogue with regulatory agencies, and internal task force reported here.

## Drug safety data sharing

There is an increasing need for sharing proprietary data in pharmaceutical toxicology in a safe and sustainable way, not only among pharmaceutical companies, but also among a wider audience such as academic institutions and regulatory agencies. Data sharing has a clear benefit for the advancement of science and is reflected in numerous collaborative data sharing initiatives (e.g. IMI eTOX, ^1^ IMI eTRANSAFE, ^2^ BioCelerate ^3^).

Effective sharing of drug safety data will require a widely adopted policy framework that addresses non-technical barriers such as organisation, trust and intellectual property protection. Regulatory acceptance of the results of drug safety models as part of the new drug application (NDA) submission process requires consultation and collaboration with regulatory bodies to develop accepted standards and documentation. The eTRANSAFE Consortium, aims to develop data sharing and knowledge management guidelines and policies necessary for industry and other organisations to share drug safety data by consulting with different stakeholders (regulators, industry, scientific bodies) to ensure these guidelines are widely adopted, and recognised by relevant policy makers such as the International Council for Harmonisation of Technical Requirements for Pharmaceuticals for Human Use ( ICH), Clinical Data Interchange Standards Consortium ( CDISC), or the Organisation for Economic Co-operation and Development ( OECD).

Sharing of proprietary data in precompetitive projects is increasingly common. The Innovative Medicines Initiative ( IMI) is an important driver that contributes to this phenomenon due to its encouragement of sharing drug research and development in the pharmaceutical domain. The European Medicine Agency’s Big Data Strategy ^4^ also encourages collaboration with academic research in regulatory science. The FDA’s Predictive Toxicology Roadmap, ^5^ highlights the need for effective data sharing including sharing of proprietary and sensitive data. Furthermore, NOT having FAIR (findable, accessible, interoperable and reusable) research data is estimated to cost the European economy at least 10.2 billion Euros every year. ^6^ However, data sharing in the pharmaceutical domain has been associated with risk proportional to the amount of the data shared. Efforts have been put forward in various initiatives to overcome the so-called *prisoner’s dilemma* of sharing consortia (i.e., for each individual participant, it pays to bring in as little data as possible as the risk is proportional to the volume of the shared data), although it has been exemplified in the UK Biobank ^7^ that sharing of large datasets can bring mutual benefit to the members of the consortia and should be seen as the norm for the field.

The EMA Big Data Strategy was initiated to address the challenges posed to our regulatory model by the rapidly evolving scientific landscape and technical innovation which, while bringing opportunities, also reveals uncertainties across the product life cycle. Pre-authorisation uncertainties arise for a number of reasons but include the fact that many non-clinical models fail to mirror human diseases, the small number of subjects in rare disease space, the unknown generalisability of randomised controlled trials and increasing complex trial designs. While some of these issues may be solved by more complementary evidence, the regulatory acceptability of this is far from certain. Post-authorisation, regulatory initiatives to increase reporting of adverse reactions has been very successful with over 1.5 million reports a year coming into EudraVigilance from centrally authorised drugs alone. ^8^ However, identifying a true signal within the noise is challenging, exemplified by the fact that from more than 1.5million reports in 2018, only 3.4% resulted in a validated signal, ^8^ but linkage of multiple data sets and types may provide insights into the underlying causes and molecular biological mechanisms.

Multiple factors influence the safety such as co-medications and co-morbidities and genomic and lifestyle together drive individual metabolic variability and susceptibility. Regulators will increasingly need to capture a more holistic picture with a temporal span of an individual. This requires data sharing at a scale and scope currently not available. Phase I of the EMA Big Data Strategy ^9^ highlights the strategic importance of data sharing to stakeholders and its regulatory impact and sets out the elements that will be required to enable regulatory acceptance of the evidence derived from such activities. The challenge is significant; data is dynamic, heterogeneous, complex and layered with relationships which need to be preserved. Moreover, many public databases are siloed and utilise different terminologies often with local modifications. The overarching recommendation from the Big Data Taskforce ^9^ centres around the need for global, harmonised, comprehensive and open data standards to facilitate an understanding of data quality, drive interoperability and thus enable linkage of data, all of which will support regulatory acceptability of the derived evidence. It is a vision of the EMA Big Data strategy that data sharing needs to happen within a sustainable framework, and the strategy identifies support mechanisms to promote the culture of data sharing which is mutually beneficial for all stakeholders. Fundamental to any data sharing activities must be the establishment of robust and comprehensive data protection processes to fully comply with obligations under the General Data Protection Regulation (GDPR). To see broad adoption by external stakeholders of the recommendations of the EMA Big Data Taskforce, it needs to provide i) demonstrable individual and global benefits to promote adoption (ensuring sustainability with clear returns on investment), ii) agile guideline development to support innovation (adaptability), iii) validation of mapping and maintenance of these mappings in line with clearly defined regulatory agency requirement.

Following the mandate of the FDA Predictive Toxicology Roadmap, coupled with the Critical Path Initiative established in 2004 aiming to modernise drug development research, ^10^ the FDA Office of Clinical Pharmacology has initiated the PredicTOX project ( https://c-path.org/programs/predictox/) with the aim to build a computational analysis platform to model molecular mechanisms underpinning drug-induced cardiotoxicity. The project introduces a governance structure that includes the role of a third-party *honest broker* by the C-Path Institute that transparently facilitates the interactions among the public, private, and regulatory stakeholders. The C-Path Predictive Safety Testing Consortium (PSTC) is the initiative to collaborate with the PredicTOX project on biomarker’s validation and safety with the vision of “safety as the gold standard” for translational drug safety. The pilot study of PredicTOX is founded on building the molecular model for cardiotoxicity associated with tyrosine kinase inhibitors in cancer patients. Key questions emerging from PredicTOX include researchers' access to patient-level data, and addressing data standards beyond SEND/SDTM. ^11^ The functional modules of PredicTOX are divided into tasks: i) data collection from pharmaceutical partners, ii) data curation, standardisation, and integration, iii) querying tools for building predictive computational models. Critical planning for such integration and tooling focuses on reusing and adapting existing databases and simulation tools. An application ontology ^12^ (i.e., *the PredicTOX Ontology*) is undergoing planning and development to drive the integration and querying of data in PredicTOX to address the semantic infrastructure needed to combine data beyond those coded within SEND/SDTM.

## eTRANSAFE recommendations

### Overcoming challenges in aggregating translational data

The overarching policies for the eTRANSAFE consortium highlighted some of the most commonly encountered challenges, and revealed solutions established within eTRANSAFE and by different research consortia. The recommendations provide precedence that can be used to overcome company internal resistance to data sharing and knowledge management. This will include a survey of the GDPR challenge, and the needs of data protection and data access procedures to manage the new legal and regulatory requirements.

These solutions are the shared foundation for the eTRANSAFE guidelines for data sharing and knowledge management in the following areas:

1) *Data gathering* - The different vocabularies and standards used in eTRANSAFE need to be normalised to a central identifier mapping system e.g., compound structure with InChI, IUPAC names, ^13^ or ChEMBL/ChEBI identifiers, ^14^ or adverse event with MedDRA vocabulary to aid linking to OBO Foundry ontologies. ^15^ The project-specific central identifier mapping system is the foundation for the eTRANSAFE application ontology.
2) *Data curation* - When data enter the eTRANSAFE data warehouse, the quality assurance and control (QA/QC) process is performed via clean-up and curation to ensure that each data entry is properly catalogued with a suitable identifier tag and label to ensure the FAIRness ^16^ of the data
3) *Data integration* - Sensible data integration pipelines should include considerations on how data are going to be (re-)used for analysis in the eTRANSAFE platform, and possibly by other secondary analyses outside the consortium.
4) *Reuse of original data and secondary processed data* - Reusability is determined by metadata design, development, and implementation driven by end-user scientists to ensure that the right data can be found with the right metadata associated to them. As technology for data generating and storage evolves, data management for reuse requires consideration of the method and technology to preserve the data and their related processing provenance. This is critical in the drug safety regulatory domain where provenance and traceability of data records need to be preserved for future reference.
5) *Data processing/recall timeliness* - Drug safety surveillance is monitored in real-time. Therefore, collecting and analysing information of all data points that support the findings of real-time adverse event reporting in a timely manner is needed. Identifying a responsive data integration model that answers to this real-time monitoring remains a critical. Minimizing the latent lag time between the data integration pipeline spectrum and response time to the public health monitoring is a priority in data management.

### Capturing how data were acquired, processed, and encoded for research reproducibility

Quality control and assurance for reproducible research are key to address data reusability. The voluntary genomics data submission at the FDA ^17^ highlights challenges when processing algorithms differ between organisations and set out a framework for computational reproducibility. ^18^ This highlights the criticality of FAIR sharing, in the aspect of FAIR principles, and mutual beneficial sharing among the data-exchanging parties. It is stressed that productive sharing requires standards on samples, data, and processing protocols.

Sharing and integrating data without conventions of data curation makes the data un (re-)usable. A governance framework that describes the data lifecycle for data reuse in drug R&D is a requirement to maintain sustainability, reproducibility, and integrity of the data. The FAIR principles ^16^ provide a contextual guideline to establish FAIR data via technical implementations. Examples of emerging projects that aim to formalise these FAIR data management include the FAIRplus project, FAIRsFAIR, and ELIXIR CONVERGE. The supporting tasks for this aim also lead to the prominent development of the European Open Science Cloud ( EOSC). The community efforts for FAIR data have also led to the recognition by a large international body such as the International Standard Organisation (ISO) as demonstrated in the draft of ISO/CD 20691, the CDISC SEND standards for information sharing and set out the best-practice guidelines for the adoption of the SEND standard within the eTRANSAFE Consortium to be interoperable with organisations outside the consortium.

### Practical aspects of data sharing

The sharing of precompetitive datasets is increasingly common and increasingly encouraged by the IMI. An important driver is the necessity to access large datasets for meta-data analyses, modelling and advanced machine learning approaches. This is particularly pertinent in the field of toxicology where ‘read across’ methods are gaining in importance and formal acceptance. ^19^^,^^20^ In (bio) pharmaceutical development the EMA recently published a summary report of Phase 1 of the “HMA-EMA Joint Big Data Taskforce” ^8^ recognising the changing landscape and the need for increased collaboration in regulatory science. The subsequent Phase 2 report sets out a number of practical recommendations the EMA network could maximise the use of big data by evolving its approach to data use and evidence generation.

**Figure 1.**
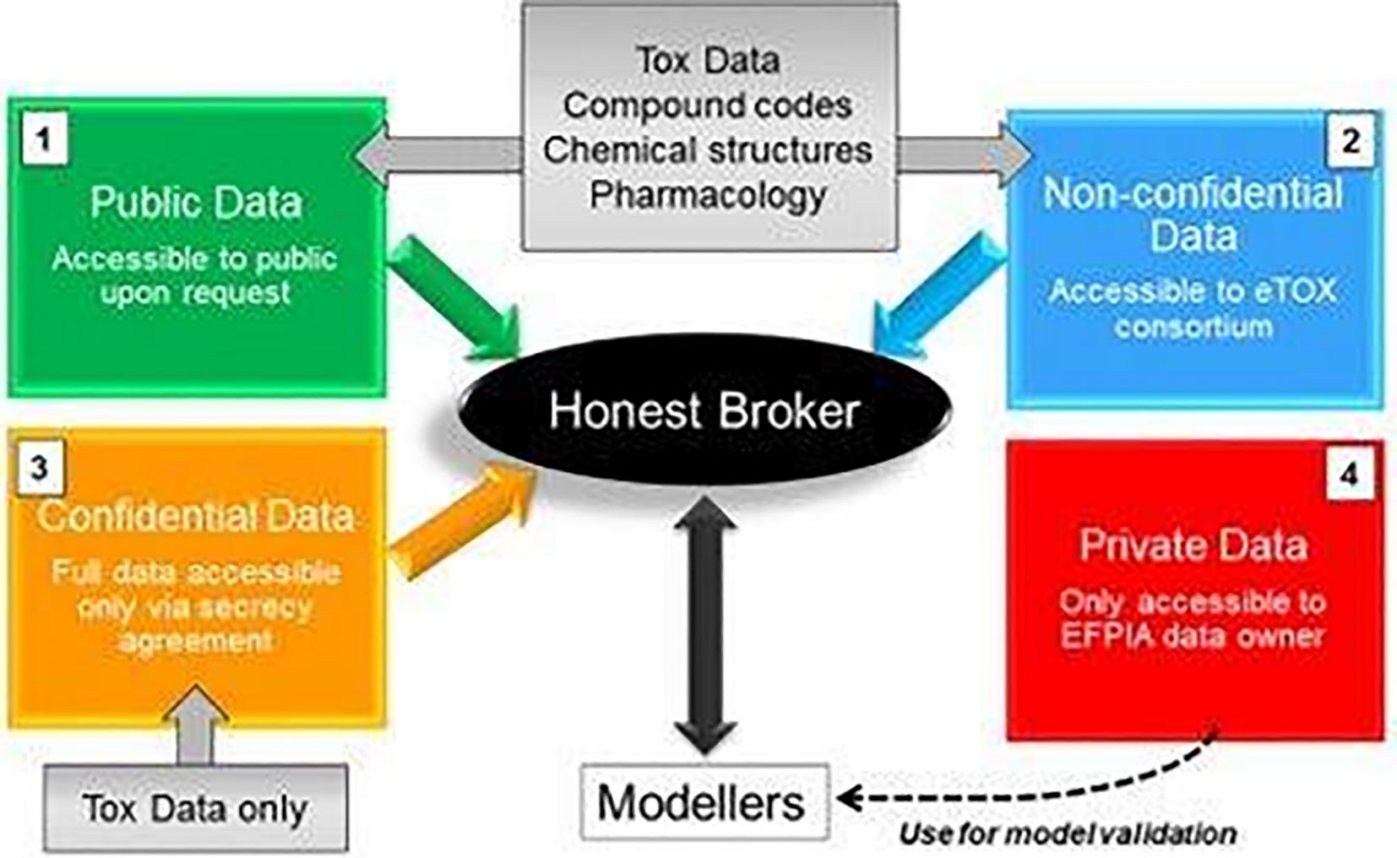
Data components, and roles of data processor. Precompetitive data shared within the eTRANSAFE Consortium are classified into public data, non-confidential data, confidential data, and private data. Shared data are shuttled across participatory partners and managed by the Honest Broker who facilitates data access and exchange across the data processors in different roles.

The eTOX project ^21^ successfully demonstrated historical toxicological data sharing among 30 public and private stakeholders. The eTOX platform deployed a computational system with integrated data to support toxicology prediction. The motivation for data sharing to lower the drug development cost and enhance the biological understanding of cross-species safety prediction provided the foundations for the establishment of eTRANSAFE. In summary, the eTOX project has overcome the challenges of data sharing public-private partnership by addressing four areas of consideration: legal boundaries, data processing technology and technical implementations, standards and vocabularies guidelines and development for data curation, and the culture of trust in sharing across the stakeholders ( Figure 1, see also ^27^).

The eTRANSAFE incorporates human clinical outcomes into the data pool, thus introducing challenges of working out the difference between aggregate versus individual level data. Clinical data are very diverse and are reported at aggregate/population level (e.g., literature, drug labels) or individual level (e.g., spontaneous reports, Electronic Health Record extraction, insurance claims), and others may come in a combination of both aggregate and individual level (e.g., clinical trials, disease registries/cohorts). Access authorisation to each source needs to be clearly indicated - e.g., sharing of individual level data are never permitted for EHR, while literature or disease cohort reporting can be shared with the right access authorisation. Furthermore, the requirements of each data processor’s roles have to be identified to set the interaction boundaries across the different roles based on their aspect of interest. Regulatory agencies may require sharing of human clinical data without exposing the individual identities, but at the same time the cohort interpretation remains meaningful for regulatory use as outlined in the EMA guideline for clinical data publication. This in its requirements poses a challenge that remains to be resolved. ^22^ Funders and publishers may require that cohorts be constructed to be made sharable. These requirements, in turn, encourage private-sector industry partners to open up and share data with authenticated parties on a case-by-case question-specific basis. Additional incentive for the originator data-donating patient subject may be through an explicit statement of their rights to be contacted should their donated data lead to a new discovery that may have an effect on their health and well-being.

The practical views of the challenges as described here could only happen in a setting where there exists an *honest broker* to facilitate data sharing by acting as a trusted neutral partner hosting the shared data, and controlling data access in accordance with the wishes of all the data donors (i.e., the EFPIA partners at the consortium) while data remain the intellectual property of the donors. As *trust* is identified as one of the key challenges in data sharing, it is important that the honest broker possesses the quality of transparency - the broker is known to some (if not all) partners, the broker is neutral with no conflict of interest, and that the broker is secure. It is therefore easier to establish trust when the honest broker is represented by a not-for-profit organisation. Further incentives for data donors to trust the honest broker may also be seen where the broker can contribute the added value to the shared data, e.g., enhancing the quality of the data through data curation or analysis to find new insights. To answer the challenges previously described in data gathering (individual partner’s legal approval, GDPR, potential misuse of data), data processing and harmonisation, technical aspect to implementation and security, rules of engagement, and sustainability are summarised here.

It is recommended that a common legal agreement to obtain data from data donors should be established with high-level management based on decision criteria that all partners agree to, and avoid needing, to get individual approval on a case-by-case basis. The efficiency of this approach is achieved by simplifying the approval process. Once data are successfully obtained in the gathering step, it is important to establish an agreed definition of data views and confidentiality. Note that it must be determined how data can be used and shared prior to the gathering process so that informed consent and GDPR transparency are possible. The consolidation of views and relevance indications are not identified up-front sometimes, and thus may need a data curation protocol that lays down the methods to define vocabulary/numerical unit standardisation, format normalisation, error catching and QA/QC of the data, granularity of the data, and explicit terms of use. The data that are considered valuable will be driven by the use cases. It is important to note that use cases may evolve over time, and the curation protocol may need to be reviewed periodically. Considerations in the curation protocol lead to recommendations that the curation pipeline needs to be implemented in a way that data curation can be automated as much as possible, and errors of data entry are caught early in the process. Defining granularity of the data clarifies what the integration can achieve in terms of aggregating raw data that is an irreversible process, i.e., aggregated data of an averaged calculation value cannot be de-aggregated to single individual data points. Once data are gathered and processed for sharing, security of the shared data has to be planned in detail. The security controls put in place need to be appropriate and be aligned with the expectations of the data donors. The role of an honest broker also covers the ground of ensuring that rules of engagement are set in place for the governance of data donation from partner, decision making process, or exceptions of circumstances for example.

Key determination of sustainability leads to the question of reproducibility over time. As the honest broker holds a unique position in data handling for all partners, the broker can also facilitate discussions around long-term sustainability. It is only sensible that data are sustained beyond the project lifetime but this is only practical if the value of the data and the number of continuing data users/interested parties exceeds the cost of maintenance and support for training and updates. It is a feedback loop that if data are kept but not updated, they will rapidly lose their value. However, data may differ in different versions of release, querying dynamic data to perform the same analysis may yield different results thus losing the reproducibility. Software used in data analysis, if not regularly updated, will become outdated and incompatible over time. Additionally, if confidential data are used to build and/or validate in silico predictive model these data cannot be shared, leaving exclusively the predictive models that are shareable. Without data governance and provenance for the supporting data, these predictive models are not sustainable. Investigation of reusable workflows such as those of the Common Workflow Language and containerisation ^23^^–^^25^ to take a *snapshot* of the data and their accompanied analysis software used in a window of time is underway as a possible solution to this sustainability and reproducibility challenge.

## Discussion

There are other critical data management considerations in the drug R&D and regulatory domain that remain unanswered challenges. Long-term preservation has been previously addressed by a time-limitless storage of original data as exemplified in the FDA Sentinel system ^26^ that retains all raw data of the studies with regulatory status, but this approach is now proving more difficult as data volume is growing exponentially at a faster rate these days. Version indicator of a dynamic dataset will fail to reproduce as some context of use will always need exact results. Understanding the meaning of data is crucial to understanding the process of the toxicology model validation framework to be confident of the clinical endpoints. One-size-fits-all validation method is implausible, so the FDA calls for a fit-for-purpose validation of the model. This is a paradigm shift from outcome-oriented validation to process-centric validation.

The fit-for-purpose metadata validation is also required for combining data encoded with different standards. Finding the right standards and tools requires that metadata of the resources are searchable and can be linked with other resources in the integrative framework. Schematic mark-up of the data and tooling resource metadata is critical to this process. To date, establishment of Schema.org and Bioschemas are seen as the foundational model for the schematic mark-up approach for the findability of FAIR-data movement.

The “context of use” of the data in data sharing and knowledge management when interacting with the regulatory partners should also be discussed, clarified, and established as early as possible in the process in the current emerging landscape of “quantitative regulatory science”. Early discussion with the regulatory partner(s) on specific research objectives to define governance processes will aid the collaboration in the pre-competitive data sharing space. This requires active engagement with all pharma stakeholders. This requirement, in turn, highlights a challenge in overcoming concerns of competitive advantage across stakeholders to promote understanding of the value of sharing, incentive and benefit, and to alleviate fears of data misuse. It is also important to note that the modern quantitative regulatory science will need to address the response to drug monitoring in real-time. Data sharing process will have to account for response timeliness. As data are streaming into the analysis pipeline in (close to) real-time, it is a goal of regulatory actors to also respond in a timely manner to any potential safety signals.

Consideration of data availability and governance continues past the project end date. Project sunsetting at the legacy stage needs planning that addresses the data use and access after the project lifetime. Organisation of data access committee and establishment of data access policy need to be set up before the project end date, preferably, early in the project life cycle. If subjects are not told about the ‘afterlife’ of their data, they may believe their information will only be used over the course of the project. Planning for legacy data may be difficult where it is not clear whether or how this legacy phase may be funded, but researchers should consider the likelihood that any large-scale collection of biomedical research, obtained at public expense and generating published findings, data may be archived for further use. While it is possible for data to be kept for ‘longer periods’ for research and archiving under the GDPR, this retention must still be done transparently, and in a way subjects would reasonably expect.

Consolidation and addressing all requirements of eTRANSAFE data sharing and knowledge management for sustainability and research reproducibility highlights the challenges in the domain of legality, standards & technology, data, institutional culture, and organisational governance. By the completion of eTRANSAFE, the Overarching Policies workgroup aims to deliver a comprehensive data sharing and knowledge management plan that accounts in the different aspects of knowledge management and can sustain over time.

## Data availability

No data is associated with this article.
