# Supplementary material for: eTRANSAFE: Building a sustainable framework to share reproducible drug safety knowledge with the public domain
Source: F1000Res. 2022 Mar 7;11:ELIXIR-287. [Version 1] doi: 10.12688/f1000research.74024.1 (PMC9096149; doi:10.12688/f1000research.74024.1)
Supplement: Peer review history

# Peer review: Reviewer response for version 1

**DOI:** 10.5256/f1000research.77731.r127597

**Published:** 2022-05-11

**Authors:** Ishwar Chandramouliswaran

**Affiliations:** 1 Office of Data Science Strategy, National Institutes of Health, Bethesda, MD, USA

This article surveys the current landscape and value of broader FAIR-sharing of drug safety data to allow reuse of of the data from across many different projects to enable new discovery, possible only with such aggregation of data. The eTOX example nicely demonstrates incentives for sharing and reuse of such pre-competitive data for new discovery.

 The article provides best practice recommendations/solutions to consider at each stage of the research lifecycle - data gathering, its curation and integration, and subsequent reuse/secondary analysis by a third-party. It might help to also consider targeting these as actionable tasks to the relevant stakeholders.

 The concept of an honest broker is a good one and it might help to also consider including characteristics for one and the stakeholder(s) that could best serve in this role. The authors can consider using the example of C-PATH's PSTC and penciling what worked and identifying what could be improved to ultimately better define the skills/expertise required of such an 'honest broker'.

Is the work clearly and accurately presented and does it cite the current literature?

Yes

If applicable, is the statistical analysis and its interpretation appropriate?

Not applicable

Are all the source data underlying the results available to ensure full reproducibility?

Partly

Is the study design appropriate and is the work technically sound?

Yes

Are the conclusions drawn adequately supported by the results?

Yes

Are sufficient details of methods and analysis provided to allow replication by others?

Yes

Reviewer Expertise:

Biomedical informatics products and services management/development.

I confirm that I have read this submission and believe that I have an appropriate level of expertise to confirm that it is of an acceptable scientific standard.

# Peer review: Reviewer response for version 1

**DOI:** 10.5256/f1000research.77731.r127011

**Published:** 2022-03-29

**Authors:** Merran Smith

**Affiliations:** 1 Population Health Research Network, University of Western Australia, Perth, WA, Australia

**Sarntivijai *et al*, eTRANSAFE article review 28 March 2022**

 This article identifies a framework to share drug safety knowledge with the public domain, recognising that drug safety data are drawn from many different sources which are covered by different governance requirements and metadata rules. 

 It describes lessons learnt from the eTRANSAFE Consortium’s efforts to integrate drug safety research at scale as well as recommendations to support eTRANSAFE guidelines for data sharing and knowledge management. These cover data gathering, curation, integration, reuse and processing.

 Key challenges and work to date to address them are described, including the additional complexity associated with use of human clinical data. Recommendations include use of a trusted ‘honest’ broker to facilitate data access.

 Figure 1 identifies the types of pre-competitive data to be shared with the Honest Broker in the eTRANSAFE consortium. It is an important diagram and it would be helpful to include a sentence or two about the Figure/model in the body of the article, especially with respect to any integration and use of private data.

 Overall, this is a valuable article which summarises challenges and identifies recommendations related to sharing of reproducible drug safety knowledge.

 Some parts of the paper could be more clearly written to highlight the importance of the work. For example, the Abstract, Paragraph 2, first sentence and the last sentence in the paper would benefit from rewording.

 With respect to availability of ‘source data underlying the results’, this is a descriptive paper outlining development of a framework for sharing of drug safety knowledge. The rationale for the framework is well presented and related, underpinning work is referenced. Details of activities undertaken to develop the framework are not included and would be beyond the scope of the paper.

Is the work clearly and accurately presented and does it cite the current literature?

Yes

If applicable, is the statistical analysis and its interpretation appropriate?

Not applicable

Are all the source data underlying the results available to ensure full reproducibility?

Partly

Is the study design appropriate and is the work technically sound?

Yes

Are the conclusions drawn adequately supported by the results?

Yes

Are sufficient details of methods and analysis provided to allow replication by others?

Yes

Reviewer Expertise:

Health information management and development

I confirm that I have read this submission and believe that I have an appropriate level of expertise to confirm that it is of an acceptable scientific standard.
